# Antimicrobial Resistance and Sports: The Scope of the Problem, Implications for Athletes’ Health and Avenues for Collaborative Public Health Action

**DOI:** 10.3390/antibiotics13030232

**Published:** 2024-02-29

**Authors:** Ognjen Ožegić, Branka Bedenić, Sunčanica Ljubin Sternak, Mario Sviben, Jasminka Talapko, Iva Pažur, Ivana Škrlec, Ivan Segedi, Tomislav Meštrović

**Affiliations:** 1 Department of Anaesthesiology, Intensive Medicine and Pain Management, Sestre Milosrdnice University Hospital Center, 10000 Zagreb, Croatia; ognjen.ozegic@kbcsm.hr (O.O.); iva.pazur@kbcsm.hr (I.P.); 2 Faculty of Kinesiology, University of Zagreb, 10000 Zagreb, Croatia; ivan.segedi@kif.unizg.hr; 3 Medical Microbiology Department, School of Medicine, University of Zagreb, 10000 Zagreb, Croatia; bbedenic@kbc-zagreb.hr (B.B.); sljsternak@stampar.hr (S.L.S.); mario.sviben@hzjz.hr (M.S.); 4 BIMIS-Biomedical Research Center Šalata, School of Medicine, University of Zagreb, 10000 Zagreb, Croatia; 5 Department of Clinical and Molecular Microbiology, University Hospital Centre Zagreb, 10000 Zagreb, Croatia; 6 Clinical Microbiology Department, Teaching Institute of Public Health “Dr Andrija Štampar”, 10000 Zagreb, Croatia; 7 Parasitology Department, Microbiology Service, Croatian National Institute of Public Health, 10000 Zagreb, Croatia; 8 Faculty of Dental Medicine and Health, Josip Juraj Strossmayer University of Osijek, 31000 Osijek, Croatia; jtalapko@fdmz.hr (J.T.); iskrlec@fdmz.hr (I.Š.); 9 University Centre Varaždin, University North, 42000 Varaždin, Croatia; 10 Institute for Health Metrics and Evaluation, University of Washington, Seattle, WA 98195, USA; 11 Department for Health Metrics Sciences, School of Medicine, University of Washington, Seattle, WA 98195, USA

**Keywords:** antimicrobial resistance, antibiotics, *Staphylococcus aureus*, MRSA, sports, athletes, surveillance, health promotion, public health, global health

## Abstract

Antimicrobial resistance (AMR) poses a global threat, leading to increased mortality and necessitating urgent action—however, its impact on athletes and the world of sports has hitherto been neglected. Sports environments (including athletic and aquatic) exhibit high levels of microbial contamination, potentially contributing to the spread of resistant microorganisms during physical activities. Moreover, the literature suggests that travel for sports events may lead to changes in athletes’ gut microbiomes and potentially impact their antibiotic resistance profiles, raising questions about the broader implications for individual and public/global health. The prevalence of *Staphylococcus aureus* (*S. aureus*) among athletes (particularly those engaged in contact or collision sports) ranges between 22.4% and 68.6%, with MRSA strains being isolated in up to 34.9% of tested individuals. Factors such as training frequency, equipment sharing, delayed post-training showers, and a history of certain medical conditions are linked to higher colonization rates. Moreover, MRSA outbreaks have been documented in sports teams previously, highlighting the importance of implementing preventive measures and hygiene protocols in athletic settings. In light of the growing threat of AMR, there is a critical need for evidence-based treatment guidelines tailored to athletes’ unique physiological demands to ensure responsible antibiotic use and mitigate potential health risks. While various initiatives—such as incorporating AMR awareness into major sporting events—aim to leverage the broad audience of sports to communicate the importance of addressing AMR, proactive measures (including improved AMR surveillance during large sporting events) will be indispensable for enhancing preparedness and safeguarding both athletes’ and the general public’s health. This narrative review thoroughly assesses the existing literature on AMR and antibiotic usage in the context of sports, aiming to illuminate areas where information may be lacking and underscoring the significance of promoting global awareness about AMR through sports.

## 1. Introduction

Antimicrobial resistance (AMR) has emerged as one of the principal causes of mortality on a global scale [1,2], resulting in the increasing impetus to implement national action plans against this health hazard [3]. Despite substantial efforts being made through various international initiatives over the past decade to curb the spread of resistant agents [4], a grim outlook persists. Estimations report 1.27 million annual deaths attributed to AMR (10% of these being in Europe), as well as a troubling projection of this number escalating to 10 million by 2050 [1,2,5]. Likewise, infections as a whole pose a considerable burden on athletes, leading to significant morbidity and a considerable amount of time being lost for sports activities [6,7]. Paradoxically, the critical issue of AMR remains markedly underacknowledged and underexplored in the context of sports.

This inaugural narrative review in the field offers a comprehensive assessment of the current literature on AMR within the realm of sports, which is a field that has been hitherto neglected in both research and public health initiatives. Our aim was to appraise the overall issue of resistant microorganisms in the sports environment and microbiomes of athletes, with a special focus on methicillin-resistant *Staphylococcus aureus* (MRSA) infections as an exemplar of infections resistant to treatment in athletes. Furthermore, we explored the possible outcomes of dealing with resistant pathogens, especially when the selection of antibiotics involves medications that could negatively impact the health of athletes. Additionally, we also wanted to shed light on the imperative task of raising global awareness about AMR through sports and sporting events.

## 2. Antimicrobial Resistance in Sports Environment and Athletes’ Microbiomes: Current State of the Art

It is known that athletic facilities and gymnasiums harbor diverse microbial populations, with the existing literature suggesting varying levels of air contamination—up to 1.02 × 10^3^ bacterial colony-forming units per cubic meter (CFU/m^3^) and up to 1.44 × 10^2^ fungal CFU/m^3^ [8,9]. Regarding bacterial contamination on surfaces, concentrations of up to 3.7 × 10^3^ CFU/cm^2^ were noted [9,10]. This is problematic, as during activity and physical exertion, the rate of inhalation surpasses normal levels, leading to an elevated rate of inhalation/exhalation of bioaerosols [11]. Additionally, factors such as a comparatively high density of individuals in these venues, confined spaces, inadequate ventilation systems and heightened human activities within indoor sports facilities influence indoor air quality [12,13].

Still, the exploration of AMR and the existence of resistant strains within athletic environments remains an area of limited research and understanding. One notable contribution was a research endeavor pursued in Poland, showing that 73% of all *Staphylococcus* strains found in the airborne bioaerosols of sports facilities were resistant to benzylpenicillin, also harboring a small rate of resistance to gentamicin, levofloxacin and rifampicin [14]. However, the problem becomes particularly noteworthy given the widespread utilization of disinfectants within these venues, which is a factor that adds complexity to the dynamics of AMR in these settings and underscores the need for comprehensive investigations that delve into the interplay between disinfectant practices and the development of resistance.

Several research groups were intrigued by this very question, utilizing a genetic vantage point to try to obtain some answers. Hartmann et al. [15] discovered that dust samples collected from multifunctional facilities (used for both sports and education purposes) exhibited a median reads per kilobase per million reads (RPKM) value of 23.24 per sample for antimicrobial resistance (AMR) genes. Among these genes, the most prevalent were extended-spectrum beta-lactamase *bla*_SRT-1_, tetracycline resistance *tet(W)* and macrolide resistance *erm(B)* genes. Notably, a correlation of disinfectant triclosan usage with efflux pump *tet(K)* and 23S rRNA methyltransferase *erm(X)* genes was observed, which confer resistance to tetracyclines and macrolides, respectively [15]. Similarly, there was a correlation between the disinfectant methylparaben and the *cmr*, *erm(c)* and *erm(33)* efflux pump genes, which result in macrolide resistance. Even though these findings did not suggest that triclosan and methylparaben contributed to the survival advantage of bacteria carrying AMR genes, Mahnert et al. [16] proposed a different perspective. More specifically, their metagenomic analysis revealed reduced bacterial diversity and elevated AMR levels in areas rigorously maintained for cleanliness. In a way, exposure to cleaning agents prompts bacteria to express more virulence factors, including among them those that code for resistant phenotypes [16].

Akin to that, Fahimipour et al. [17] established a link between increased usage levels of triclosan and triclocarban in operating indoor athletic venues in the United States and an increased presence of bacterial species/genera harboring miscellaneous drug resistance genes in the dust indoors. Facilities that were included in the study were public recreation centers, martial arts venues, private fitness clubs, and yoga and dance studios. Consequently, the emerging potential of bacterial resistance to penicillin, tetracyclines and macrolides has been assessed by subjecting strains obtained from dust to aforementioned antimicrobial agents/drug classes [17].

Indeed, microbial communities present in dust from athletic facilities had a significant degree of heterogeneity, with a core group consisting of six representatives: *Massilia* spp., *Pseudomonas* spp., *Cutibacterium* (*Propionibacterium*) *acnes*, *Enhydrobacter aerosaccus*, *Subdoligranulum* spp. and C2-like viruses [17]. The composition of dust predominantly reflected contributions from the skin and urogenital communities of those using surveyed athletic venues. Furthermore, 28.3% of the isolated colonies demonstrated resistance to at least one of the drugs appraised in the study, and these drug-resistant bacteria were found in nearly all surveyed venues—suggesting widespread distribution across facilities [17]. Some of the drug-resistant bacteria exhibited resistance to multiple antimicrobial compounds, with additional biocide tolerance. The species with the strongest resistance profile, *Micrococcus luteus*, frequently displayed quinolone and aminoglycoside resistance phenotypes via genes associated with protecting DNA gyrase or encoding membrane protease activity, respectively.

Leonard et al. [18] conducted a study to assess the potential human exposure to resistant strains of bacteria during recreational water activities in England and Wales. Their main focus was to assess the prevalence of extended-spectrum beta-lactamase (ESBL)-producing *Escherichia coli* (*E. coli*) resistant to third-generation cephalosporins (3GC) (i.e., *bla*_CTX-M_-bearing *E. coli*) in coastal water samples. They integrated data on the volume of water ingested during various water sports (such as swimming, diving, surfing, boating and wading/splashing), and subsequently reported the mean number of 3GC-resistant ESBL-producing *E. coli* ingested during each water-related activity. Notwithstanding a low prevalence of resistance to 3GC in *E. coli* (0.12%), there is an evident risk for individuals that are engaged in professional or recreational water activities [18]. More specifically, over 6.3 million water sport sessions in 2012 resulted in the ingestion of at least one strain of *E. coli* that was resistant to 3GC. It is important to note that this study focused solely on resistance to this class of antibiotics, suggesting that the actual recreational exposure to AMR bacteria may be underestimated.

Even travel to a specific sporting event can present a risk for colonization by resistant microorganisms. A recent study—which used 16S rRNA amplicon sequencing on fecal samples—showed how the journey to the Republic of India made by Irish cricketers (as a lead up to the 2016 World Cup) resulted in a profound impact on the microbial constituents of their gut microbiome [19]. This temporal shift was accompanied by changes in the presence of both antibiotic resistance and virulence genes. In two athletes, there was a notable elevation in the number of identified antibiotic resistance genes in fecal samples from the post-travel period. Specifically, before the India travel episode, the antibiotic resistance landscape remained individual and specific to both of these athletes; however, in the aftermath of their journey, there was a discernible increase in the potential for resistance to eight antibiotic classes observed in both of them [19]. These findings underscore the dynamic nature of the gut microbiome in response to travel and emphasize the intricate interplay between external factors, such as geographical location and the microbial components within the human body [20]. The increased potential for antibiotic resistance raises questions about the implications of travel-related changes in gut microbiota on individual athlete health and performance, but also for the broader landscape of antibiotic resistance.

## 3. Methicillin-Resistant *Staphylococcus aureus* as a Paragon in the Field of Research

*S. aureus* is known to cause skin and soft tissue infections, which can lead to unfortunate consequences in athletes—not only due to pain, discomfort and the potential of progressing to more serious illness, but also due to the fact that these infections can lead to delays in training, drop outs from athletic tournaments, etc. Taking such observations into consideration, it should not come as a surprise that *S. aureus* is the most commonly researched microorganism in sports. It is a widely known fact that MRSA can be especially pathogenic; accordingly, many researches try to further elucidate the exact proportion that this strain accounts for in the total number of *S. aureus* isolates. This knowledge can be useful in order to predict outbreaks, isolate those at risk and promptly act upon these findings. Consequently, a literature review was conducted in order to provide data on MRSA isolation rates among various sports (Table 1).

Mascaro et al. [21] conducted a cross-sectional study analyzing the prevalence of *S. aureus* carriage and antibiotic resistance among athletes participating in contact or collision sports in Italy. In their research, 42% out of 238 participants were identified as *S. aureus* carriers. Furthermore, colonization was associated with training frequency, sports equipment sharing, delaying showering after training and a previous history of sinusitis, pharyngitis and skin manifestations. In their study, Mascaro et al. found that 1.3% of all participants carried MRSA, and that MRSA accounted for 3% of all *S. aureus* isolates [21].

Likewise, Couvé-Deacon et al. [22] performed a cross-sectional study exploring *S. aureus* colonization among French athletes at risk of infection caused by community-acquired MRSA. Out of 300 athletes included in their study, 183 of them were carrying *S. aureus*, and 1 study participant was carrying MRSA. The authors determined that the sports at risk of community-acquired (CA) MRSA infection were weightlifting, rugby, wrestling, fencing, martial arts and sports that involved a ball (i.e., volleyball, football, basketball, baseball or handball).

Besides that, Couvé-Deacon et al. [23] published another study on the outbreak of Panton–Valentine leukocidin (PVL)-associated *S. aureus* infection in a rugby team in France. They found that 68.6% of 51 team members were colonized with methicilin-sensitive *S. aureus* (MSSA), but no MRSA strains were identified on that occasion. In order to halt the occurrence and spread of skin abscesses caused by *S. aureus* on team members, the authors implemented decontamination and hygienic precaution protocols on several occasions.

Furthermore, Rackham et al. [24] studied the community-acquired MRSA (CA-MRSA) nasal carriage rate among collegiate athletes. Altogether, 1.8% of 277 participants were identified as CA-MRSA carriers, although no data on the total number of *S. aureus* isolates have been disclosed. The authors highlight that, in their study, a relatively low MRSA nasal carriage rate was observed—akin to that in the general population. They attribute this finding to the institutions’ awareness of the CA-MRSA control and prevention recommendations proposed by the Centers for Disease Control and Prevention (CDC) in the United States.

Moriya et al. [25] screened the nasal carriage of an *S. aureus* USA300 clone (a highly pathogenic CA-MRSA clone) among asymptomatic teammates of a collegiate football player with serious complications caused by that microorganism. The results indicated that 5.8% of 69 nasal samples were identified as containing CA-MRSA strains, and three of those actually represented a USA300 clone. The authors provided no information on the number of total *S. aureus* isolates.

A study conducted in northern Taiwan by Wang et al. [26] on college student athletes and the control group (consisting of non-athletes) showed that 22.4% of 259 nasal samples were positive for *S. aureus*, and 1.54% were identified as containing MRSA. Of those, 139 were athletes and had the same rate of nasal carriage of MSSA (21%) as that of the control group. The rate of nasal MRSA carriage in the group comprising only athletes was 0.72%. Moreover, Braun and associates [27] conducted a study on CA-MRSA incidence among high school and intercollegiate athletes in the USA during two time periods. The incidence rate of infection was 15.5 per ten thousand athletes and 16.3 per ten thousand athletes in 2012–2013 and 2013–2014, respectively.

Champion et al. [28] studied the prevalence of MRSA isolates from healthy collegiate athletes participating in nine different sports during a 12-week period. The samples were collected from nose, axillae and inguinal regions, and a detailed microbiological analysis was performed. Of the 223 participants, 34.9% tested positive for MRSA (139 total MRSA isolates). The authors identified a strong correlation between the MRSA carriage rate and wrestling/baseball. Furthermore, Lear and associates [29] conducted research on the incidence of nasal colonization with *S. aureus* and soft tissue infection in high school athletes. The study enrolled 190 participants, among which 44 (23.16%) were colonized with MSSA, and none had MRSA. The authors report ten skin and soft tissue infections, but these were all attributed to bacterial and fungal causative agents.

In addition, Oller et al. [30] intended to determine the presence of *S. aureus* in samples derived from footballers and wrestlers, as well as from their surrounding environments. Human-body-derived samples showed that the *S. aureus* rate was 6% in the control group consisting of on-campus non-athletes (N = 50, no MRSA isolates), 47% in football players (N = 70, with 7 or 10% of them harboring MRSA), and 64% in wrestlers (N = 25, out of which 1 or 4% had MRSA). To further elucidate these findings, the total number of athletes was 95 (70 football players and 25 wrestlers), and MRSA was identified in 8 (8.4%) of them.

Garza et al. [31] conducted their research on CA-MRSA in a professional football team. They obtained nasal samples from 108 players and staff members, and found that none were carrying MRSA. However, 26.8% of nasal cultures tested positive for MSSA. On the other hand, Stevens et al. [32] performed screening on a female collegiate basketball team after one of the players presented with a CA-MRSA skin and soft tissue infection, as a frank surveillance effort. Of the 13 team members that were subjected to testing, MRSA was isolated in the nasal swabs from 4 of them (30.7%). The authors emphasize the necessity of considering CA-MRSA as a pathogen in cases presenting with soft tissue infections.

In their retrospective analysis, Romano et al. [33] examined the outbreak of CA-MRSA among collegiate football team players in a period of three years. They found that complicated skin and soft tissue infections were present in 1.8% of 107 participants in the year of 2002, in 15.8% of 107 players in 2003, and in only 1 individual (0.96%) out of 104 subjects in 2004. During the period of study (three years), 14 cases of CA-MRSA were established (4.4% of the 318 total participants).

Creech et al. [34] conducted surveillance of CA-MRSA nasal colonization and skin/soft tissue infection over the period of one year in 126 student athletes participating in lacrosse programs, monitored separately as male and female teams. For the male team, nasal colonization with MRSA exhibited significant variability throughout the athletic season, ranging from 4% during the low season to a peak of 19% during the high season [34]. However, no association was found between the increase in colonization and skin/soft tissue infections. In the women’s lacrosse team, MRSA nasal colonization rates varied from 11% to 23%, depending on the season [34].

Stacey at al. [35] conducted research in the year of 1996 on MRSA in a rugby team consisting of 20 players. Five of the players tested positive for MRSA (25%) after presenting with cutaneous infection that could not be successfully cured with standard beta-lactam antibiotics. Seven other team members were further found to be carrying the MSSA strain. The authors believe that this is the first report of an MRSA infection outside of a hospital setting.

Rihn et al. [36] reported on a CA-MRSA outbreak in a high school football team. The samples were taken from 102 subjects in total (90 players and 12 staff members); of those, 3 (2.9%) tested positive for MRSA. Moreover, 32 of 102 samples (31.4%) were identified as containing MSSA. The authors did not provide information on whether positive isolates belonged to players or members of staff. It was also observed that the majority of infections occurred on the extremities, these being more prone to skin injury. Kazakova et al. [37] investigated an outbreak of MRSA-related abscesses among professional football players. They found that 8.6% of 58 players encountered an MRSA infection. They linked the infection to a certain position, and to a higher body mass index (BMI). All the MRSA strains contained the gene for PVL, as well as the USA300 gene complex.

Archibald et al. [38] searched for connections between MRSA infections in a team of collegiate football players and the risk factors. Nasal cultures were obtained from 109 athletes and 38 staff members. The results showed that 3.7% of the athletes and 7.9% of the staff were positive for MRSA isolates. Also, 24 isolates (16%) were MSSA. The authors suggest that the higher prevalence of infection was found among junior players without established hygiene practices, as well as those sharing personal hygiene accessories at home.

Sutton et al. [39] conducted an investigation on the successfulness of the implementation of anti-MRSA strategies among collegiate football players, particularly in regards to CA-MRSA. Out of the 25 sampled football players, MRSA cultures were positive on at least one site in 15 players (60%). According to the study, the suggested strategies to mitigate such high carriage rates include wound covering, better hygiene practices, avoiding equipment sharing and also encouraging athletes to report any skin lesions to their coaches.

Zaborova et al. [40] conducted research on staphylococcal skin flora in water sports athletes. The authors conducted this study by using four groups (modern pentathlon, athletic swimming, synchronous swimming and water polo), comprising 51 athletes in total. *S. aureus* was isolated in 54.7% of the 51 athletes. The authors identified MRSA in 13.8% of synchronous swimmers and 10.3% of pentathlon athletes. No MRSA strains were identified among swimmers and water polo players.

Huijsdens et al. [41] researched an outbreak of CA-MRSA among members of a soccer team and their close contacts. In total, 42 soccer club members and 14 of their roommates were screened. Of those, nine players (21.4%) and two roommates (22.2%) were positive for MRSA infection. The authors of the study note that the pathogen spread within and across teams. They conclude that this was the first report of a CA-MRSA ST80-IV strain outbreak in an athletic team.

## 4. Implications of Antimicrobial Resistance for Antibiotic Treatment: Is It Time for Evidence-Based Guidelines?

In order not to miss their training schedule and to maintain athletic performance, athletes are more prone to consuming antibiotics in comparison to the general population [42,43]; therefore, the rise in AMR may significantly impact the selection of antibiotics. Studies have shown that intensive exercise for long durations increases the risk of upper respiratory tract infection, frequently necessitating the use of antimicrobial agents [42,44], and that drug choice may be influenced by the presence of MRSA in the upper respiratory tract (although the mentioned pathogen rarely causes respiratory infections per se).

Antibiotics can be classified performance-wise as bactericidal or bacteriostatic, as well as chemically into groups of beta-lactams, aminoglycosides, tetracyclines, fluoroquinolones, macrolides, carbapenems and trimethoprim (among others) [42]. Although generally considered safe, antibiotics as a group of many classes of drugs with a common therapeutic goal share their burden of unwanted effects being manifested on the human body [43,45,46].

Adverse effects are numerous and are generally associated with specific groups of antibiotics that are now prescribed pervasively when faced with *S. aureus* and other bacterial species resistant to many narrow-spectrum antibiotics (Table 2). For example, aminoglycosides are linked to problems such as headache, paraesthesias, vertigo, rash and dizziness [43], whereas tetracyclines display unwanted effects in terms of manifestations of teeth discoloration, enamel hypoplasia, diarrhea, photosensitivity, appetite issues, mouth/lips sores and swelling of the tongue [44]. The use of fluoroquinolones can come with the burden of digestive disorders, blood sugar issues, cardiac arrhythmias with heart-rate corrected QT interval (QTc) prolongation in the electrocardiogram (ECG), detachment of the retina, tendinopathy or tendon rupture, peripheral neuropathy and aortic aneurysm [46]. Macrolides, on the other hand, carry the potential of adverse effects manifesting as allergic reactions, cholestatic hepatitis, cardiac arrhythmias and transitory auditory impairment [47]. The use of carbapenems can lead to gastrointestinal disorders, seizures and allergic reactions [48], while the use of trimethoprim can lead to itching and rashes, gastrointestinal symptoms, changes in taste, headache, skin sensitivity to the exposure to sun, swollen tongue and fever [49].

Some of the abovementioned unwanted effects that can arise from the use of antibiotics can be especially problematic for the athletes. A narrative review by Odorici et al. [50] showed that the use of different tetracycline-class antibiotics in usual doses leads to diverse photosensitive reactions in patients after they are exposed to sunlight, such as sunburns, photosensitivity and phototoxicity [50]. A recent study by de Castro-Maqueda [51] showed that athletes who practice water sports professionally spend around four hours a day exposed to sunlight. This is concerning, as studies found that almost half of the athletes performing outdoor sports do not wear sunscreen at all [52].

Another potentially serious side effect that can occur when using fluoroquinolones and macrolides is an increased risk of cardiac arrhythmias (QTc prolongation; ventricular arrhythmias), which can sometimes even be fatal [45]. Furthermore, QTc interval prolongation induced by antibiotics can lead to disqualification in athletes with congenitally prolonged QTc intervals. More specifically, aforementioned antibiotics can elevate the QTc interval to above the upper limit during routine electrocardiography screening, which is frequently executed prior to some organized competitive sports events [45,53,54,55].

Broad-spectrum antibiotics are often used in order to cover a wider range of infections caused by both Gram-positive and Gram-negative bacteria, and are more commonly used when faced with the issue of AMR. As mentioned before [42,46], fluoroquinolones are commonly associated with an increased risk (i.e., three to six times) of developing inflammation of tendons and Achilles tendon rupture [56]. The other factors that contribute to Achilles tendon rupture during the use of fluoroquinolones include age over 60, male gender, renal disease, physical activity and concomitant steroid use [46,57,58]. In their publication, Lewis and Cook conclude that athletes, trainers and medical staff should perhaps discontinue fluoroquinolones in an event of tendinopathy [46].

Diarrhea that can arise due to the use of antibiotics is a common adverse reaction to those drugs. Rates differ from 5% to 39%, depending on the studies [45]. Symptoms may start immediately upon the commencement of treatment or can be delayed by a few weeks, and are most commonly preceded by the use of broad-spectrum antibiotics [45]. It is known that even a simple case of diarrhea can have a significant adverse effect on the performance and overall health status of an athlete [59].

Maier et al. also found that nonsteroidal anti-inflammatory drugs (which are pervasive in sports) can inhibit the growth of at least one strain of a microorganism, implying that non-antibiotic medications might promote the rise in AMR as well. Such a notion highlights the possibility of the future discovery of more specific drug–microbiome relationship examples, revealing potential adverse effects on athletes’ overall health [60].

Given the rise in resistant microorganisms, but also the inherent complexities and potential health risks associated with antibiotic usage in athletes, there is a pressing need for the development and implementation of evidence-based guidelines. These guidelines should consider the unique physiological demands of athletes, the specific challenges posed by their training regimens and the potential impact of antibiotics on both health and performance. However, they should also emphasize judicious antibiotic-prescribing practices to curb the development of AMR. Such evidence-based recommendations can help guide healthcare professionals, athletes, trainers and medical staff in making informed decisions about antibiotic choices and the dosage and duration of treatment, and can also serve as a crucial tool to promote responsible antibiotic use. This dual focus on athlete health and the larger societal impact underscores the importance of evidence-based practices in guiding antibiotic usage within the sports community.

## 5. Raising Global Awareness of Antimicrobial Resistance through Sports, Sporting Events and in Athletes

The intersection of sports and the global awareness of AMR underscores the crucial role that sports can play in fostering a broader understanding of this pressing global health issue. As athletes and sporting events command significant attention and influence across diverse communities, leveraging this platform to raise awareness about AMR can be used as a powerful tool for both education and advocacy. This was also recognized by the World Health Organization, which endorsed the integration of AMR awareness into globally recognized sporting days and campaigns in their recent report following global consultation meetings on AMR awareness raising [61]. More specifically, their proposal involves highlighting the issue of AMR during major international sporting events, such as the World Cup football (soccer) tournament; additionally, there is a suggestion to introduce new awards related to AMR, akin to those awarded at prestigious events like the Oscars or Olympics [61]. Finally, creating competitions that showcase the positive contributions of AMR champions is also encouraged [61]. All these initiatives aim to leverage the broad audience and attention garnered through prominent global events to effectively communicate the importance of addressing AMR.

Not surprisingly, countries around the globe have already undertaken incremental advancements in that direction. One notable example comes from Spain, where numerous universities have decided to actively participate in commemorating European Antibiotic Awareness Day, a European health initiative that is coordinated by the European Centre for Disease Prevention and Control (ECDC) [62]. Aligned with the initiative of the Ministry of Health in Spain within the National Plan against Antibiotic Resistance, universities that are involved decided to collaborate to host an event known as ‘Corre sin resistencia’ (‘Run without resistance’) [63]. This collective effort involves organizing a non-competitive five-kilometer race simultaneously in multiple universities across Spain, encouraging individuals of all ages to either run or walk, promoting awareness about antibiotic resistance [63].

Another noteworthy example involves Ace Africa Kenya, which secured generous support from ReAct/South Centre Switzerland and Jane Bubear Sports Foundation to promote World Antimicrobial Awareness Week initiatives in 2020 [64]. This included a community football tournament and various local events in Siaya County, located in the western part of Kenya. In conjunction with the football tournament, residents of Siaya County (both children to adults) actively participated in awareness-raising endeavors during World Antimicrobial Awareness Week [64]. The multifaceted approach encompassed educational sessions, banners, booklets, specialized drama performances and featured segments on two local FM radios—effectively disseminating crucial information on AMR to the general public [64]. This comprehensive project succeeded in reaching over 250,000 children and adults [64], not only enhancing their knowledge regarding health, hygiene and the intricacies of AMR, but also best practices associated with antibiotic usage.

Numerous athletes who have successfully overcome MRSA infections share their inspirational stories, serving as motivation for others, but also as figures of caution about the perils of AMR [65]. Case reports raising awareness of this problem have also been published in the medical literature. A salient example is a case report by Yokomori et al. [66] that describes a life-threatening incident involving a 20-year-old Japanese university athlete infected with a CA-MRSA USA300 clone harboring PVL [66]. The individual in question had sustained multiple abrasions on his limbs while playing rugby, and the authors posit a strong likelihood that the particular USA300 clone responsible for this case originated from the nose of one of his fellow teammates [66]. Such a clone was already known as a significant threat among the professional players of American football [37].

However, besides raising awareness, there is a crucial necessity for an improved AMR surveillance system to be used during big sporting events. A notable example of proactive measures was observed during the Fédération Internationale de Football Association (FIFA) World Cup 2022 hosted by Qatar. The host country embraced meticulous wastewater surveillance with a propensity to detect resistant (and emerging) pathogens, as well as genetic AMR signatures [67]. Although no specific report on AMR has been published following this approach, the adoption of such measures could empower countries hosting large-scale sporting events and global health authorities to actually monitor and understand the patterns of AMR transmission. This, in turn, would significantly contribute to enhancing preparedness and implementing proactive prevention and control plans to safeguard not only athletes’ health, but also the health of the general public [67]. In addition, such integration of advanced surveillance mechanisms in the context of sports events aligns with the broader global diagnostic efforts to address the multifaceted challenges posed by AMR.

Such integrative efforts are especially pertinent in low- and middle-income nations, where the challenge of AMR is exacerbated (primarily due to the initially high burden of infectious diseases) [1,68], and which also witness an increasing number of mega-sporting events being awarded to them [69]. Here, lessons from other mass gathering events can be used as a blueprint to inform future strategies on big sport events. For example, it is well known that huge religious events such as Kumbh Mela in India, Hajj in Saudi Arabia, Arba’een in Iraq or Grand Magal of Touba in Senegal can generate optimal conditions for the transmission of a vast array of microorganisms, contaminating the environment, surfaces, food, water and human skin [70]. Consequently, this leads to the potential spread of various pathogens (including multidrug-resistant bacteria) among attendees, local residents and organizers [70,71]. Exact examples are, therefore, now slowly being integrated into national policies and agendas to guide planning and preparedness strategies based on evidence. Nevertheless, this raises the question of why comparable approaches have not been seen in terms of large-scale sporting events.

Thus, beyond promoting a multidisciplinary approach that acknowledges the interconnectedness of individuals and their shared surroundings, organizations and global stakeholders should prioritize the critical necessity for increased political and financial investments in AMR research during large-scale sport events, which have hitherto been unjustly overlooked. This includes emphasizing the importance of understanding the dynamics of AMR and allocating appropriate resources in the context of all types of events that pose infection and AMR hazards—from smaller indoor tournaments to mass sport events. Collaborative efforts should encompass a wide spectrum of initiatives, with sporting events being integral to both the global AMR research agenda and broader action plans aimed at averting this hazard. Athletes, as public figures, can serve as ambassadors in communicating the importance of responsible antimicrobial use, the consequences of misuse and the collective efforts needed to combat the rise in resistant microorganisms.

## 6. Conclusions

The convergence of sports and global AMR awareness presents a unique opportunity to educate and advocate, as envisioned in the proposal of the World Health Organization (WHO) to integrate AMR awareness into major international sporting events. Primary strategic goals in national action plans center on enhancing the comprehension and consciousness of AMR. The overarching aim is to promote sustainable investments in the field, knowledge acquisition through robust research and surveillance, lower infection rates and the optimized usage of antimicrobials [72,73]. Sporting events, particularly large-scale gatherings, offer ample opportunities to achieve at least some of those objectives—particularly to disseminate information, promote good hygiene practices and engage the public in conversations about the implications and dire consequences of AMR.

Our review shows how athletic facilities can harbor diverse microbial populations with a plethora of drug-resistant strains, posing a potential risk of coming into contact with AMR genes during physical activities. Limited research also suggests how AMR genes can be ubiquitous in dust samples, emphasizing the need for comprehensive investigations and heightened awareness in sports environments. Of course, athletes can be colonized with MRSA strains, presenting risks to fellow athletes (especially in contact sports). However, despite the commendable initiatives, there is still a dire need for enhanced AMR surveillance during sports events, urging increased political and financial investments in research and mitigation strategies across various events. This is exactly where athletes can play an indispensable role as ambassadors for responsible training and judicious antimicrobial use. By intertwining sports with global AMR awareness, there is a potential to reach diverse demographics and inspire collective action toward a healthier and more sustainable future—not only for athletes and sports enterprise, but also the global community as a whole.

## Figures and Tables

**Table 1:** A comprehensive overview of methicillin-resistant *Staphylococcus aureus* (MRSA) prevalence in various sports based on a literature search.

Authors	Study Year	Sports Included	Number of Subjects	MRSA Prevalence
Mascaro et al. [21]	2019	taekwondo, judo, karate, wushu/kung fu, boxing, kick-boxing, mixed martial arts (MMA), Muay Thai, football	238	1.3%
Couvé-Deacon et al. [22]	2017	rugby, wrestling, basketball, volleyball, handball, fencing, martial arts, football, weight-lifting, baseball	300	0.33%
Couvé-Deacon et al. [23]	2016	rugby	51	0%
Rackham et al. [24]	2010	football, baseball, soccer, track, wrestling, basketball, dance/cheer, golf, volleyball, rowing, swimming, softball, diving	277	1.8%
Moriya et al. [25]	2020	football	72	5.8%
Wang et al. [26]	2017	soccer, basketball, taekwondo, judokas, tennis, table tennis	139	0.72%
Braun et al. [27]	2016	football, wrestling, soccer, hockey, baseball, indoor track, lacrosse, basketball, cheerleading, cross country, outdoor track, volleyball	per 10,000	0.155%; 0.163%
Champion et al. [28]	2014	wrestling, baseball, tennis, softball, track, basketball, lacrosse	223	34.9%
Lear et al. [29]	2011	football	190	0%
Oller et al. [30]	2010	football, wrestling	95	8.4%
Garza et al. [31]	2009	football	108	0%
Stevens et al. [32]	2008	basketball	13	30.7%
Romano et al. [33]	2006	football	318	4.4%
Creech et al. [34]	2010	football, lacrosse	126	4–19% in men; 11–23% in women
Stacey et al. [35]	1998	rugby	20	25%
Rihn et al. [36]	2005	football	102 (90 players and 12 staff members)	2.9%
Kazakova et al. [37]	2005	football	58	8.6%
Archibald et al. [38]	2008	football	109	3.7%
Sutton et al. [39]	2014	football	25	60%
Zaborova et al. [40]	2011	modern pentathlon, athletic swimming, synchronous swimming, water polo	51	13.8% (synchronous swimmers); 10.3% (pentathlon athletes)
Huijsdens et al. [41]	2006	soccer	42 (players)	21.4%

**Table 2:** An overview of adverse reactions associated with different drug classes in athletes.

Antimicrobial Drug	Adverse Effects	Reference
Carbapenems	Allergic reactions Gastrointestinal disorders Neurological disorders	[48]
Trimethoprim	Allergic reactions Anorexia Dysgeusia Cephalodynia Hyperpyrexia Gastrointestinal disorders Skin sensitivity to solar exposure Glossitis	[49]
Macrolides	Allergic reactions Cardiac arrhythmias Transitory hearing impairment Biliary hepatitis	[47]
Fluoroquinolones	Gastrointestinal disorders Ablation of retina Tendinopathy and tendon rupture Blood glucose metabolism disorders QTc prolongation Cardiac arrhythmia Aneurysm of aorta Peripheral neuropathy	[46]
Beta lactams	Allergic reactions Gastrointestinal disorders	[47]
Aminoglycosides	Skin rash Ataxia Vertigo Hyperpyrexia Paresthesia Cephalodynia Kidney failure	[43]
Tetracyclines	Gastrointestinal disorders Teeth discoloration Photosensitivity Anorexia Mouth sores Glossitis	[44]
